# How to switch a master switch

**DOI:** 10.7554/eLife.01159

**Published:** 2013-07-30

**Authors:** Christopher Hein, Alfred Wittinghofer, Volker Dötsch

**Affiliations:** 1 **Christopher Hein** is at the Institute of Biophysical Chemistry and the Buchmann Institute of Molecular Life Sciences, Goethe University, Frankfurt, Germanyhein@bpc.uni-frankfurt.de; 2 **Alfred Wittinghofer** is at the Max Planck Institute of Molecular Physiology, Dortmund, Germanyalfred.wittinghofer@mpi-dortmund.mpg.de; 3 **Volker Dötsch** is an *eLife* reviewing editor, and is at the Institute of Biophysical Chemistry and the Buchmann Institute of Molecular Life Sciences, Goethe University, Frankfurt, Germanyvdoetsch@em.uni-frankfurt.de

**Keywords:** cell signalling, nucleotide exchange factor, diacylglycerol, Human

## Abstract

The crystal structure of a nucleotide exchange factor in white blood cells reveals an autoinhibitory mechanism that reinforces the switch-like behaviour of the signalling protein Ras.

**Related research article** Iwig JS, Vercoulen Y, Das R, Barros T, Limnander A, Che Y, Pelton JG, Wemmer DE, Roose JP, Kuriyan J. 2013. Structural analysis of autoinhibition in the Ras-specific exchange factor RasGRP1. *eLife* **2**:e00813. doi: 10.7554/eLife.00813

**Image** A linker region between two domains of RasGRP1 (red) blocks the Ras binding site in the absence of calcium ions and diacylglycerol (not shown)

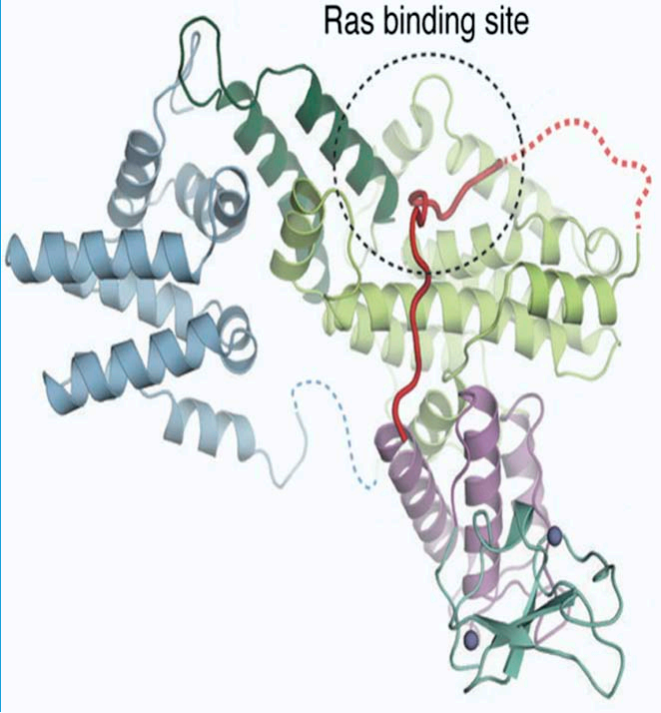

Cells in many organisms can modify their behaviour after receiving signals from elsewhere in the organism. In principle, these signals can evoke two types of responses: a linear response or a switch-like response. The regulatory circuits that control various metabolic pathways within the body often involve linear responses: in other words, the strength of the response depends on the strength of the input signal. However, the signalling pathways that regulate the proliferation, death or survival of cells often involve switch-like responses: that is, there is no response when the input signal is below a certain threshold, and there is a full response when the input is above this threshold.

One prototypical switch in the cell is a small protein called Ras that can exist in two different conformations (shapes), depending on whether it is bound to the regulatory small molecules GTP or GDP (Vetter and Wittinghofer, 2001). When it is bound to GTP, Ras is active and can interact with many other proteins. However, when Ras is bound to GDP it is inactive. The ability of Ras to exist in either of two different states is a prerequisite for its role as one of the master switches of the cell and, consequently, mutations in Ras have been found in many cancers.

However, this capacity of Ras to cycle between ‘on’ and ‘off’ states is not sufficient to build a switch because, in theory, the relative amount of activated Ras could be proportional to some incoming signal. To make Ras a true switch, other regulatory proteins are necessary. Indeed, an entire arsenal of different proteins and mechanisms has been found to control the activity of Ras (and other, related, proteins), thus ensuring that signalling by Ras is tightly regulated under normal conditions. Now, in *eLife*, a collaboration between the laboratories of John Kuriyan, of UC Berkeley, and Jeroen Roose, of UC San Francisco, reports new insights into one of these regulatory proteins, a factor important for the development of white blood cells (Iwig et al., 2013).

Two categories of proteins can regulate Ras (Figure 1). GAPs (GTPase activating proteins) help to make Ras inactive by accelerating the conversion of GTP into the related compound GDP. In contrast, GEFs (guanosine-nucleotide exchange factors) catalyse the replacement of GDP by GTP, and thus activate Ras (Bos et al., 2007). These GAPs and GEFs must also be tightly regulated to ensure that Ras does not go rogue: in particular, they may be restricted to certain tissues or types of cells. Kuriyan, Roose and colleagues—including Jeffrey Iwig, Yvonne Vercoulen and Rahul Das as joint first authors—focus on one such tissue-restricted GEF. Called RasGRP1, this GEF cooperates with a GEF called SOS (Son of sevenless) to regulate the activity of Ras during the development of white blood cells (Dower et al., 2000; Kortum et al., 2011).

**Figure 1.**
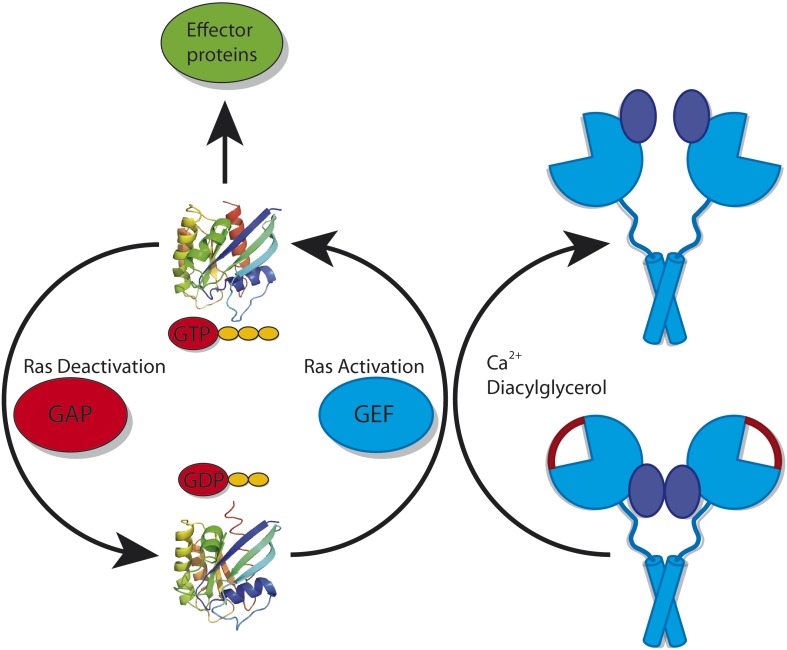
Ras cycles between a GTP-bound state (top; left panel) in which it interacts with downstream effector proteins, and a GDP-bound state (bottom) in which it is inactive. The activity of Ras is tightly regulated by many different proteins. However, it is also important for cells to control the activity of the proteins that deactivate Ras (called GAPs) and the proteins that activate Ras (called GEFs). RasGRP1 is a GEF: in its inactive conformation (bottom; right panel) it cannot bind to Ras because the binding site for Ras is blocked by a linker peptide (depicted in red) and because the C1 domains (depicted in dark blue) that recruit it to the membrane are buried within the RasGRP1 dimer. RasGRP1 can be activated by calcium ions and diacylglycerol binding to it: this removes the linker peptide and exposes the C1 domains (top; right panel).

RasGRP1 has been found to consist of several domains. The catalytic Cdc25 domain is responsible for interacting with Ras and for enhancing the exchange of GDP for GTP. RasGRP1 also contains a C1 domain that can direct it to the membrane, where it can associate with Ras. Through another domain, the EF-hand domain, RasGRP1 can bind calcium ions, which results in significant changes in conformation. Finally, a C-terminal coiled-coil region can assist RasGRP1 in forming dimers.

It has previously been shown that SOS can be prevented from binding to Ras by an autoinhibitory mechanism, in which the Ras binding site on the catalytic domain of SOS is blocked by another portion of the SOS protein. Interestingly, the activation of SOS requires activated Ras: GTP-bound Ras can associate with an allosteric binding site on SOS that is separate from the catalytic site of the protein. This induces a conformational change in SOS that un-blocks its catalytic Ras binding site. Additional Ras can then bind to the catalytic site and, in turn, be activated by SOS. However, this presents an enigma: if activated Ras is necessary to activate SOS, how is this Ras activated in the first place? The structural and functional investigations of RasGRP1 by the UC team offer an intriguing model for how this might occur. They reveal that RasGRP1 crystallizes in a dimeric, autoinhibited conformation. The autoinhibition occurs because the linker peptide that connects the Cdc25 catalytic domain with the EF domain obstructs the Ras binding site.

But this is only one aspect of inhibition. As has been seen in the autoinhibited structures of many other proteins (Sicheri and Kuriyan, 1997; Yu et al., 2010), autoinhibition is often achieved by more than one domain-domain interaction. These multiple autoinhibitory mechanisms have the advantage that full activation requires at least two different—and often independent—signals. With independent signals the likelihood of activation becomes the product of the likelihood of each individual activation process (Prehoda et al., 2000). Integrating two different processes thus results in the switch-like behaviour that regulates many pathways. In RasGRP1, this second level of autoinhibition is achieved by the sequestration of the C1 domain within the dimer. More specifically, the C1 domain (which directs the protein to the membrane) interacts with, and is hidden by, the catalytic Cdc25 domain and the calcium-binding EF domain.

This autoinhibited conformation of RasGRP1 also suggests how the protein could become activated. Binding of diacylglycerol to the C1 domain and calcium to the EF domain would induce large conformational changes that are incompatible with the closed, autoinhibited conformation. Functional studies carried out by the UC team have indeed shown that both calcium and diacylglycerol can activate RasGRP1. This leads to a compelling model for how the activity of Ras is regulated in developing white blood cells. Activation of RasGRP1 activates a small amount of membrane-associated Ras, which can then interact with the allosteric binding site on SOS. Activated SOS, in turn, strongly amplifies the activating signal for Ras, thus establishing the switch-like behaviour required to regulate this protein. This interplay of a weak initiator and a strong enhancer protein leads to a safe, and at the same time very effective, biological switch that ensures that white blood cell development is adequately controlled. It will be exciting to see if this model can be further verified by functional analysis of the interactions among these three proteins in cells.
